# Impulsivity and pain attentional bias in veterans receiving care for chronic non-malignant pain

**DOI:** 10.3389/fpain.2026.1652567

**Published:** 2026-04-23

**Authors:** James M. Bjork, Peter J. Norris, Zina Trost

**Affiliations:** 1 Mental Health Service, Central Virginia VA Health Care System, Richmond, VA, United States; 2 VA Mental Illness Research, Education and Clinical Center, Durham, NC, United States; 3 Institute for Drug and Alcohol Studies, Virginia Commonwealth University, Richmond, VA, United States; 4 Department of Psychological & Brain Sciences, Texas A&M University, College Station, TX, United States

**Keywords:** attentional bias, chronic pain, decision-making, impulsivity, pain care, veterans

## Abstract

**Introduction:**

A prevailing biopsychosocial model of chronic non-malignant pain (CNMP) includes psychological drivers of disability such as pain catastrophizing or negative mood symptomatology, such that preoccupation with pain or pain signals might be manifested at even the subconscious level and could also interact with trait impulsivity to prioritize immediate relief using opioids over safer pain management strategies. However, studies of impulsivity and cognitive biases in CNMP have often not controlled for current opioid use. Moreover, despite the ubiquity of CNMP in military veterans, pain attentional bias in veterans has not been explored.

**Methods:**

We administered computerized tasks to probe attentional capture by visual pain-related stimuli in signal detection tasks, myopic delay-discounting behavior and general symptomatology in *n* = 61 veterans who were receiving Veterans Health Administration (VHA) care for CNMP (CNMP Group) with no histories of chronic opioid use and in *n* = 38 veterans who reported no CNMP (Controls).

**Results:**

We replicated in veterans previous findings of significantly greater depression and anxiety and higher levels of pain-related disability and kinesiophobia, lower quality of life, and more severe delay discounting than Controls. However, the CNMP group did not show greater motoric impulsivity, nor greater attentional capture by distress-related visual stimuli or frustration tolerance in other tasks. Follow-up analyses indicated that across all participants, individual differences in attentional bias toward pain also did not relate to scores on a latent factor of mood and pain-related symptomatology. In contrast, psychological distress correlated positively with motoric impulsivity in a stop-signal task, specifically under conditions with a pain-related visual distractor, and high values of a negative mood factor showed a trend toward a correlation with more severe discounting of delayed rewards.

**Discussion:**

These data extend to veterans previous findings of greater psychological distress and delay discounting in adults with CNMP but offer only modest evidence that persons with CNMP who do not manage pain with opioids show increased attentional capture by pain-related visual stimuli.

## 1 Introduction

Chronic nonmalignant pain (CNMP) incurs over $500 billion dollars in economic loss annually in the United States (US) from reduced employment due to disability, complications with opioid use (1) and other costs related to increased depression, suicidality, and substance use disorder (SUD) (2). Moreover, use of opioid analgesics to treat CNMP is associated with additional psychosocial and economic burden (3) in that chronic opioids confer significant medical and psychiatric risks, including iatrogenic development of opioid use disorder (OUD) (4).

Improvement and patient acceptance of safer cognitive-behavioral strategies for CNMP management would benefit from a greater understanding of neurocognitive features of CNMP patients in the context of the biopsychosocial model of chronic pain. This model considers the mental aspects of the (subjective) pain experience and posits that many individuals with CNMP, especially those with histories of significant neuroticism or other affective symptomatology, are prone to catastrophizing their pain (5). This can result in a preoccupation with pain as a danger signal, increased attentional bias toward pain (6), or increased cognitive interference by pain or pain-related cues (7). These models also posit that severe mood symptoms in persons with CNMP amplify the urgency for attaining rapid pain relief, such as using opioid analgesics (8). In support of this, not only are patients with chronic negative mood symptoms or pre-existing SUD more likely to request and receive opioids as a long-term pain management strategy (9), these patients are also at increased neuro-temperamental risk for development of OUD if they are prescribed opioids (10).

Biopsychosocial models of chronic pain have been largely informed by questionnaire or interview-based metrics, wherein two factors have emerged as especially predictive of CNMP pain intensity and disability, namely pain catastrophizing and kinesiophobia (sometimes called pain-related fear) (11–13). Pain catastrophizing is characterized by a magnified perception of pain experience as well as helplessness and rumination regarding pain (14). Kinesiophobia refers to fear of pain and (re)injury due to movement (15). Both are associated with psychological distress such as depression and anxiety in CNMP, as well as avoidance behavior (13, 16–18) and potential medication consumption (19–21) that exacerbate a self-perpetuating cycle of pain and disability (22). As described below, individuals with elevated fear and catastrophic cognition consistently show avoidance of painful or potentially painful stimuli in both clinical and experimental settings, for instance, demonstrating cautious/restricted motor behavior in studies including reaching tasks (23, 24).

Although a mature literature has linked mood symptomatology to each of: development of CNMP (25), to chronic use of opioids for CNMP management (26), and to more difficult recovery from CNMP disability (27), less is known about specific *cognitive* features in CNMP. For example, several studies have shown in aggregate that CNMP participants show small-moderate deficits in executive functions like working memory compared to controls (28), with the caveat that these studies seldom controlled for mood or sleep disturbance or for analgesic medication effects. Of interest here is expanding knowledge of cognitive factors in CNMP that may be more central to core mechanisms of the biopsychosocial model, namely preferences for urgency/immediacy and attentional bias or capture toward pain related stimuli that may be drivers or consequences of mental preoccupation with pain. For example, preference for immediate pain relief may further stem from an interaction between elevated negative affect and subconscious preoccupation with pain with a tendency to prefer immediate gratification [as a genetically influenced (29) trait].

First, several [but not all (30)] studies have shown that compared to controls, persons with CNMP show exaggerated “delay discounting,” operationalized as a stronger preference for receiving immediate but small (usually hypothetical) rewards or losses (31) over larger but delayed rewards or losses (32, 33). In support of this idea, Dwyer and colleagues (34) recently reported that in persons in SUD recovery, severity of reward discounting correlated with pain catastrophizing. Bialaszek et al. (31) found that exaggerated discounting of losses at future timepoints was specific to chronic pain participants with high pain anxiety. Similarly, exaggerated discounting in persons with chronic pain during the COVID-19 pandemic was specific to pain participants who reported high COVID-19 related stress (33). In addition, Mistretta et al. (35) reported that decisions to defer immediate losses in favor of even larger delayed losses related specifically to pain catastrophizing. That discounting may relate more to trait-level psychopathology and less from actively experiencing pain is suggested by findings that acute laboratory administration of painful stimuli to human subjects did not change their intertemporal reward preference (36). These findings collectively suggest that strong preference for immediate rewards in some persons with CNMP is driven by high *salience* of pain to that individual, in terms of subjective emotional distress.

Second, several (but not all) studies have shown that compared to controls with no CNMP, persons with CNMP also show exaggerated attentional biases toward pain-related lexical or visual stimuli (6, 37), as a potential marker of subconscious preoccupation with pain. Attentional biases are typically indexed directly by oculomotor behavior (gaze direction and fixation) using eye-tracking or indirectly by calculating reaction time (RT) latency differences between trials with vs. without evocative stimuli in “cover” cognitive tasks. These behaviors are thought to stem from automatized subconscious processes that are difficult to feign (38). This phenomenon is akin to how persons with depression (39) or anxiety (40) show exaggerated attentional capture by visual images that have negative valence (such as sad scenes or fearful facial expressions) relative to other visual stimuli. For example, studies have shown that pain-related cues are more salient in individuals who endorse high catastrophic thinking or who show increased attentional capture by pain stimuli, such as images of people in pain (41, 42) or by objects within searchable scenes that convey pain (7). Such attentional capture has also been demonstrated among individuals who hold other distressing pain-related beliefs (such as injustice perception regarding their pain) (43). It is notable that attentional/behavioral disruption is apparent even among healthy individuals who hold threat-related beliefs regarding pain and injury. For example, among control (pain-free) participants, stimuli relating to “round-back” relative to “straight-back” lifting captured attention and lengthened reaction time (RT) in a cover task in those who held beliefs that round-back lifting damages the lower back (44). Similarly, persons with low back pain showed an implicit association between round-back lifting and danger (45). Finally, Roelofs et al. (46) used a dot probe task with images of persons conducting daily activities and found that chronic pain patients had difficulty disengaging from images of persons engaging in activities the participant previously rated as likely to cause pain.

We wished to integrate these lines of experimental evidence by collecting data on a broad range of subjective pain symptomatology and consequences (self-report questionnaires) together with objective intertemporal decision-making probes and pain attentional bias tasks and comparing these combined metrics between CNMP outpatients vs. patients with no CNMP. We also wished to determine whether pain patients with the most mood/pain-related psychosocial symptomatology also show the greatest delay discounting or attentional capture by pain-related stimuli as neurobehavioral markers of subconscious preoccupation with pain. In addition, in accordance with how the contemporary models posit that a mental schema of pain, disability, and self may overlap to amplify subjective pain experience (47), we also sought to probe implicit associations between misery and self by introducing a novel implicit association task (IAT) modified to determine whether self pronouns more readily associate with words that connotate distress vs. comfort in patients with CNMP.

In addition to deploying a broad panoply of both established and novel assessments, our study features other novel elements. First, few studies of cognitive features of CNMP have centered on veterans. This is a notable literature gap in light of their high incidence of CNMP due to the physical and psychological rigors of military life (48). In light of findings that veterans who utilize Veterans Health Administration (VHA) health care tend to have more physical and mental symptomatology than veterans who utilize private care systems (49), together with findings that persons with CNMP who seek care for CNMP tend to have greater comorbid symptomatology than those who self-manage (50), we recruited a population of veterans who were receiving VHA care for CNMP to likely attain a large range of symptomatology. Second, in light of evidence that opioid use or withdrawal can increase the severity of delay discounting (51) and degrade signal detection in attentional tasks (52), as well as have significant affective impact, we sought to avoid these effects by recruiting only veterans with no recent history of opioid use, such as prescribed analgesics, per self-report and VHA medical records review.

We hypothesized that veterans receiving VHA care for CNMP would show all of the following compared to veterans who were receiving VHA health care for other reasons (here Controls): increased psychological symptomatology in accord with previous findings, as well as increased impulsivity and greater attentional biases toward pain- or distress-related stimuli. Further, we hypothesized that, in line with predictions of contemporary models of chronic pain (47), attentional and semantic pain biases as well as decision-based impulsivity would each directly correlate with self-reported negative mood and with other pain-related psychological distress.

## 2 Methods

All recruitment and testing procedures took place at a large Veterans Affairs Medical Center (VAMC) and were reviewed and approved by the institutional review board of the VAMC.

### 2.1 Participants

Using automated VHA electronic medical records (EMR) search followed by manual chart review, we recruited two groups of veterans. The CNMP Group was composed of *n* = 61 veterans (age 45–65, mean 55.8 ± SD 6.6; 34 male) who either completed an appointment at the pain clinic at the VAMC within the past year or were slated for a pain clinic appointment within the next two months. To avoid psychomotor confounds, use of any opioids (prescription or illicit) within the past two years was exclusionary. The Control group was composed of *n* = 38 veterans (age 45–65, mean 54.2 ± 7.0; 25 male) slated for a non-pain-related appointment at the VAMC, with no evidence of chronic pain diagnostic codes, pain clinic visits or pain-related care appointments in VHA EMR. Each veteran first underwent a brief telephone interview where staff asked about any recent CNMP symptomatology or treatment for pain. Endorsement of current CNMP in prescreening in the phone screen was exclusionary for Controls.

### 2.2 Assessment session

Within a week prior to or following their appointment for clinical care at the VAMC, veterans completed a ∼100 min assessment on a Windows PC laptop. Questionnaire and behavioral responses were electronically recorded in the Inquisit 6 testing platform (Millisecond LLC, Seattle WA). Participants were paid $60 for this assessment session.

#### 2.2.1 Interview-based questionnaires of symptomatology, impairment and substance use

The first part of the assessment was composed of several questionnaires administered by a research team member in a verbal interview format. All measures utilized have been extensively used and show strong psychometric characteristics across pain-specific and non-pain populations; all measures utilized in the current study demonsrated high internal validity, with Cronbach's alpha ≥ .88.

##### 2.2.1.1 Assessments of distress

1) *Pain Catastrophizing Scale (PCS)* (14): This 13-item scale captured psychological distress stemming from experience of pain, such as feelings about pain and the patient's likelihood of remaining in pain. 2) *Tampa Scale for Kinesiophobia (TSK)* (53): On this 17-item scale, the participant rated his or her agreement on a range of 1–4 on items pertaining to concern over pain arising from certain activities (such as exercise). 3) *GAD-7* (54): This is a 7-item assessment of past-two-week symptoms of generalized anxiety, including worrying too much about different things, fear, and being on edge. 4) *Patient Health Questionnaire-9 (PHQ-9)* (55): This 9-item assessment probed the number of days (“Not at All” to “Nearly Every Day”) out of the past two weeks in which the participant has experienced symptoms of depression.

##### 2.2.1.2 Assessments of disability

*WHODAS 2.0* (56): This is a 36-item assessment of disability in several domains, including Cognition, Mobility, Self-Care, Getting Along, Household Activities, Work Activities (only assessed in the *n* = 56 participants who previously reported current employment) and Participation. 2) *PROMIS Pain Interference 1.1 (PPI)* (57): This 40-item assessment specifically probes past-week interference in life functions and activities due to experiencing pain.

##### 2.2.1.3 Assessment of substance use

*PhenX Toolkit Lifetime plus Past 30-days Substance Use:* This first probed whether the participant has ever used each of several drug classes, such as cannabis, stimulants, or sedatives. For each of those substances for which lifetime use was reported, the participant was then asked to estimate the number of days out of the past 30 that the substance was used.

#### 2.2.2 Neurobehavioral tasks

Following a 10 min break, the participant completed the following tasks intended to probe attentional bias, decision making, and subconscious association between self and misery.

##### 2.2.2.1 Signal detection cover tasks with pain-related visual distracters

###### 2.2.2.1.1 Stop signal task (SST) with pain image backgrounds

**Figure 1:**
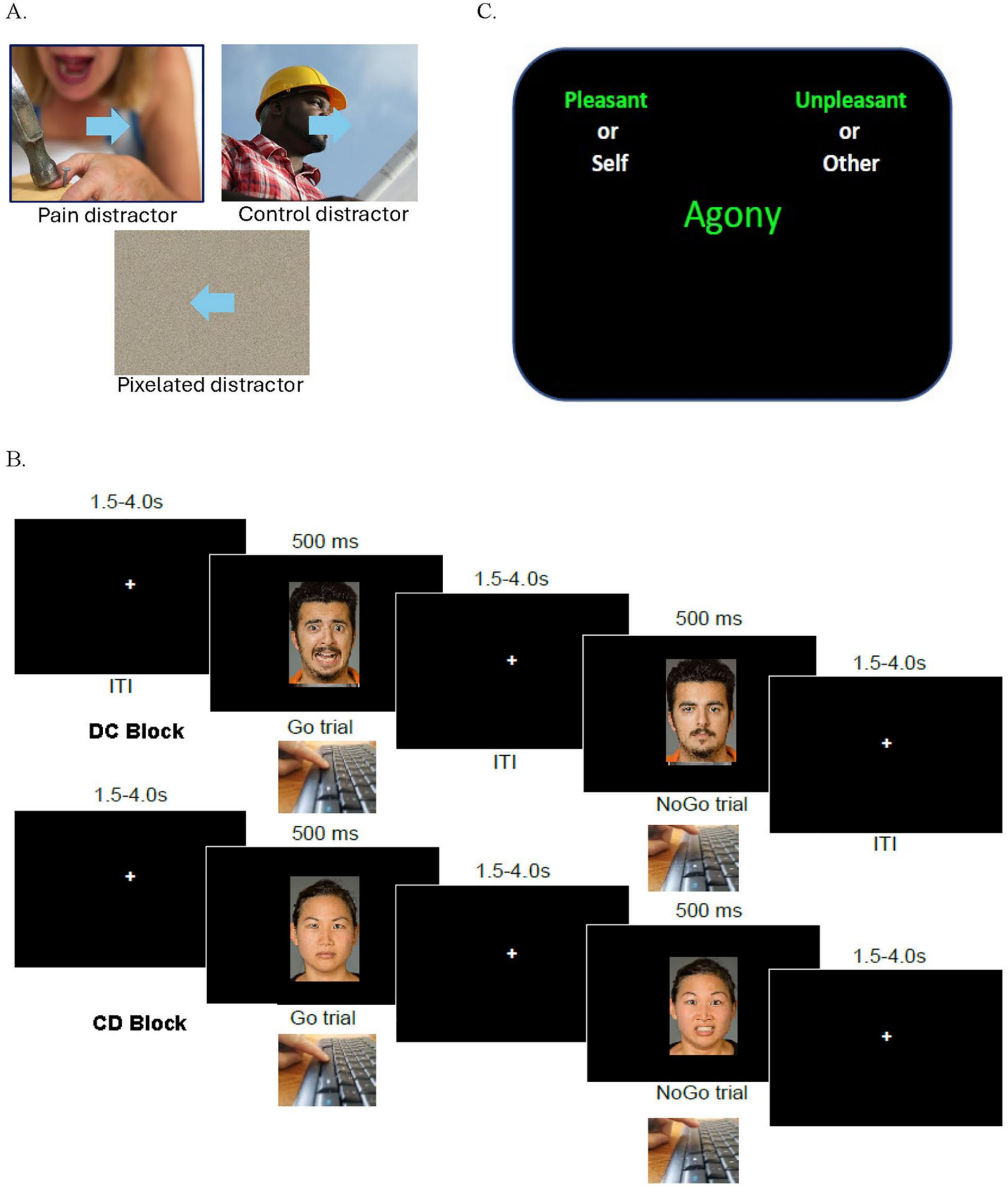
Task Diagrams. Shown are representations of each of the: **(A)** Stop Signal Task with Distractors (illustrating each distractor condition), **(B)** the Emotional Go-NoGo Task, illustrating the sequence of events for a trial in a DC block (top: respond to distressed, withhold response to calm) and trial in a CD block (bottom: respond to calm, withhold response to distressed), and **(C)** the semantic Implicit Association Task (illustrating a congruent trial where a positive word and a self pronoun are endorsed on the same side).

The SST ostensibly measures self-control, operationalized as the ability to cancel an already-initiated motor response (58). In the context of a “race” model, valid SST behavioral readouts depend on a race between motoric behavior execution processes and inhibitory processes in brain to determine whether the participant can stop a response (58). Across three, 3 min blocks (with 30 s rest breaks), the participant was asked to indicate the direction of a left- or right-pointing arrow with a left or right arrow key. On 25% of trials, the arrow changed to an upward direction with a “beep” and the participant was instructed to withhold an already-initiated motor response. The “stop-signal delay” (SSD) to this beep was continuously titrated to maintain a 50% rate of successfully stopped responses. In the “pain” task block, the background of the task changed every 12 s to a different photo of a person in pain or in an ostensibly painful posture (See Figure 1A). In the “control” task block, background images transitioned between photos of persons not in pain, and a third “pixel” block presented pixel-scrambled variants of pain and control images to match luminescence. Block order was counterbalanced between participants. Stop signal reaction time (SSRT) was the primary datum and was calculated per the integration method (59) separately for each block, where longer SSRT represents poorer ability to curtail an already-initiated response (i.e., higher motoric impulsivity), as has been found in populations with attention deficit hyperactivity disorder (ADHD) (60).

Emotional go-no-go task (EGNGT). The EGNGT is an assessment of both sustained attention and motoric self-control. In this 11 min task (61), the participant saw a series of photos of actors with either expressionless (e.g., calm) or distressed (e.g., wincing or fearful) facial expressions (Figure 1B). In one task block (“DC”), participants were to respond with the space bar to each distressed face but withhold responses to calm faces, in another task block (“CD”), the reverse instruction held. Likewise, participants completed an “HC” block wherein they were instructed to respond to happy faces and withhold responses to calm faces, and a “CH” block with the reverse instruction. RT and accuracy were compared between blocks as a function of the responding instruction. Median values of RT for each trial type were used to suppress effects of outlier long RT trials. Of interest were target RT differences and commission error rate differences within-subject as a function of block instruction. Specifically, the “salience” effect compares behavior when expression-laden faces were the instructed targets (HC, DC) in the block vs. calm faces as targets (CH, CD). The “valence” effect compares behavior when whether happy faces were either targets or non-targets (HC, CH) in the block vs. when distressed faces were either targets or non-targets (DC, CD). We specifically focused on a metric of “distress bias” calculated as median RT to distressed images when they were targets (DC) minus median RT to calm faces when distressed faces were non-targets (CD). Large RT differences between these conditions (but not between HC and CH conditions) would be suggestive of exaggerated attentional bias regarding distress-laden social information. For example, persons with opioid use disorder showed increased attentional capture by fearful faces in this task, as indicated by reduced correct responses and slower RT to fearful faces as targets (but this was not observed with regard to happy faces) (62).

##### 2.2.2.2 Decision-making tasks

Delay Discounting Task (DDT). The DDT measures decision-based impulsivity, specifically the willingness to delay gratification (63). In this 4 min task (64), the participant made several choices between earning a standard hypothetical $100 reward at various delays (by block) into the future (6 h to 5 years) vs. receiving a smaller hypothetical amount immediately. The software titrated the immediate reward amount of each choice in successive trials based on the previous choice, to arrive at the reduced subjective value of the $100 reward at each delay to receiving it. The area-under-curve (AUC) of these indifference points across delay intervals was plotted, with lower AUC values indicating greater devaluation of reward with delays (i.e., decision-based impulsivity) (65).

Frustration tolerance task. In a longitudinal study, poor frustration tolerance predicted poor translation of modest reductions in pain with rehabilitation into perceptions of better overall functioning (66). We operationalized frustration tolerance here as the incidence and timing of a decision to give up on the Behavioral Indicator of Resiliency to Distress (BIRD) task (67). It is a variant of the computerized Paced Auditory Serial Addition Task (PASAT-C) (68) originally adapted for adolescents. In this 10 min task, the participant saw a cartoon of a bird in a cage and was required to left-click a mouse arrow on an illuminated circle randomly presented within an array of 10 surrounding circles within a short time window to “free” the bird. Too-slow responses resulted in a buzz sound and distressed bird. The task first captured median RT during a 3 min practice, then applied this value as the duration window for a successful response in the next portion, so that the participant would likely hit ∼50% of targets. However, during the final “challenge” task period of interest, the allowed RT window for success was reduced to half of the duration at which the participant would succeed in 50% of trials, leading to errors on nearly every trial. The participant was invited to stop the task whenever he or she chose by clicking on a button, up to a six-minute subtask duration. Subjects completed four mood ratings (happiness, frustration, irritability, anxiety) on a Likert scale before and after the task. The choice to quit the task prematurely (binary variable) as well as the elapsed time spent in the final block (0–360 s) (69) were the key metrics of frustration tolerance. In previous work, time to endure the final block was inversely correlated with anxiety (69).

##### 2.2.2.3 Semantic attentional bias tasks

Implicit Association Task (IAT) modified with distress-related words. To potentially detect implicit associations of self with misery, we administered a 6 min implicit association task (IAT) (70) modified to present comfort- vs. pain-related words. Participants viewed a series of words in the center of the screen (Figure 1C). The participant was instructed to press a leftward or rightward key to either: a) assign a pain-related noun (e.g., “agony”) or comfort-related noun (“relax”) presented in green to either “pleasant” or “unpleasant” categories (displayed at upper right or left), or in other trials, b) assign a self-referring pronoun (“my”) or other-referring pronoun (“them”) presented in white to indicate a conceptual match with “self” and “other” categories (also displayed at upper right or left). Task blocks differed as to whether the self/other-related and comfort/pain-related category designators were assigned to the same side of the screen (R or L key). The task yields a RT-based metric “d” of bias (70) that indicates the strength of the bias wherein most individuals more readily maintain implicit associations between self and positive words (i.e., speeded RT when self-words and comfort words were endorsed on the same side of the screen relative to opposite sides). We hypothesized that in an IAT wherein negative words were pain-specific, this tendency toward self-positivity (d value) would be blunted relative to controls.

### 2.2 Data analysis

We performed independent *t*-tests to determine group-wise differences in each of total and subscale questionnaire scores, and in most behavioral task metrics. For behavior analysis in the SST and the ENGNG, which featured different conditions in different task blocks, we instead performed mixed-model repeated-measures analysis of variance, with block type as the within-subject factor, and patient group as the between-subject factor. We then correlated all questionnaire total and subscale scores with each other using Spearman rank-order correlation to minimize outlier effects. With an assumption that symptomatology scores would intercorrelate within-subject (71), we planned to reduce comparisons by conducting a principal components analyses (PCA, detailed below) of questionnaire scores to reduce multiple symptomatology assessments into a single latent factor of general distress.

## 3 Results

### 3.1 Group differences in affective and pain symptomatology

As shown in Table 1, the two study groups did not differ in age or sex representation, but the CNMP Group had significantly higher scores on every total and subscale score of symptomatology assessments compared to the Control group. Follow-up exploratory two-way factorial analyses of variance (ANOVAs) with sex and group as independent variables confirmed group-based score differences and indicated neither main effects of sex, nor any trends toward sex X group interaction effects on symptomatology.

**Table 1:** Group differences in symptomatology.

Variables	Pain Patients (*n* = 61)		Controls (*n* = 38)			
	Mean	SD	Mean	SD	*Chi-sq/t-value*	*P*
Demographics:						
Sex	34M, 27F		25M, 13F		0.982	.322
Age	55.8	6.6	54.2	7.0	1.087	.280
Interview-based Questionnaire Assessments:						
Past-30-days alcohol use (yes/no)^a^	32 Y, 28 N		24 Y, 14 N		0.917	.338
Past-30-days cannabis use (yes/no)^a^	8 Y, 53 N		8 Y, 30 N		1.105	.314
**GAD-7**	9.5	6.2	5.3	5.8	3.412	.001
**PHQ-9**	10.7	6.5	4.1	4.7	5.857	<.0001
**PROMIS: Pain Interference v1.1 Total**	126.3	39.2	60.1	29.7	9.471	<.0001
**WHODAS Cognition**	13.9	5.4	9.5	3.6	4.790	<.0001
WHODAS Mobility	14.5	5.2	7.2	2.7	9.254	<.0001
WHODAS Self-Care	7.4	3.2	4.6	1.6	5.684	<.0001
WHODAS Getting Along	10.3	5.0	7.8	4.2	2.683	.009
WHODAS Household Activities	11.8	4.5	6.3	3.1	7.167	<.0001
WHODAS Work Activities	14.8	4.9	7.7	3.2	6.552	<.0001
WHODAS Participation	22.4	7.9	13.6	7.0	5.747	<.0001
**Pain Catastrophizing Scale-Total**	25.3	14.0	8.2	8.5	7.540	<.0001
Pain Catastrophizing Scale-Rumination	9.2	4.6	3.6	3.5	6.870	<.0001
Pain Catastrophizing Scale-Magnification	5.5	3.4	1.8	2.3	6.546	<.0001
Pain Catastrophizing Scale-Helplessness	10.6	6.7	2.9	3.5	7.483	<.0001
**Tampa Scale for Kinesiophobia-Total**	46.4	7.6	37.2	7.0	6.150	<.0001
Tampa Scale for Kinesiophobia-Somatic Focus	16.8	3.8	12.0	3.5	6.446	<.0001
Tampa Scale for Kinesiophobia-Activity Avoidance	19.4	3.3	15.4	3.8	5.410	<.0001
Distress Factor (standardized factor score)	0.96	2.0	−1.57	1.2	7.780	<.0001
Mood Factor (standardized factor score)	0.45	1.3	−0.7	1.1	4.825	<.001

Metrics incorporated into principal components analysis-based Distress Factor in **bold**.

^a^ PhenX data not available for 1 participant.

### 3.2 Group differences in neurobehavioral task results

#### 3.2.1 Signal detection cover tasks with pain-related visual distracters

##### 3.2.1.1 Stop signal task (SST)

**Figure 2:**
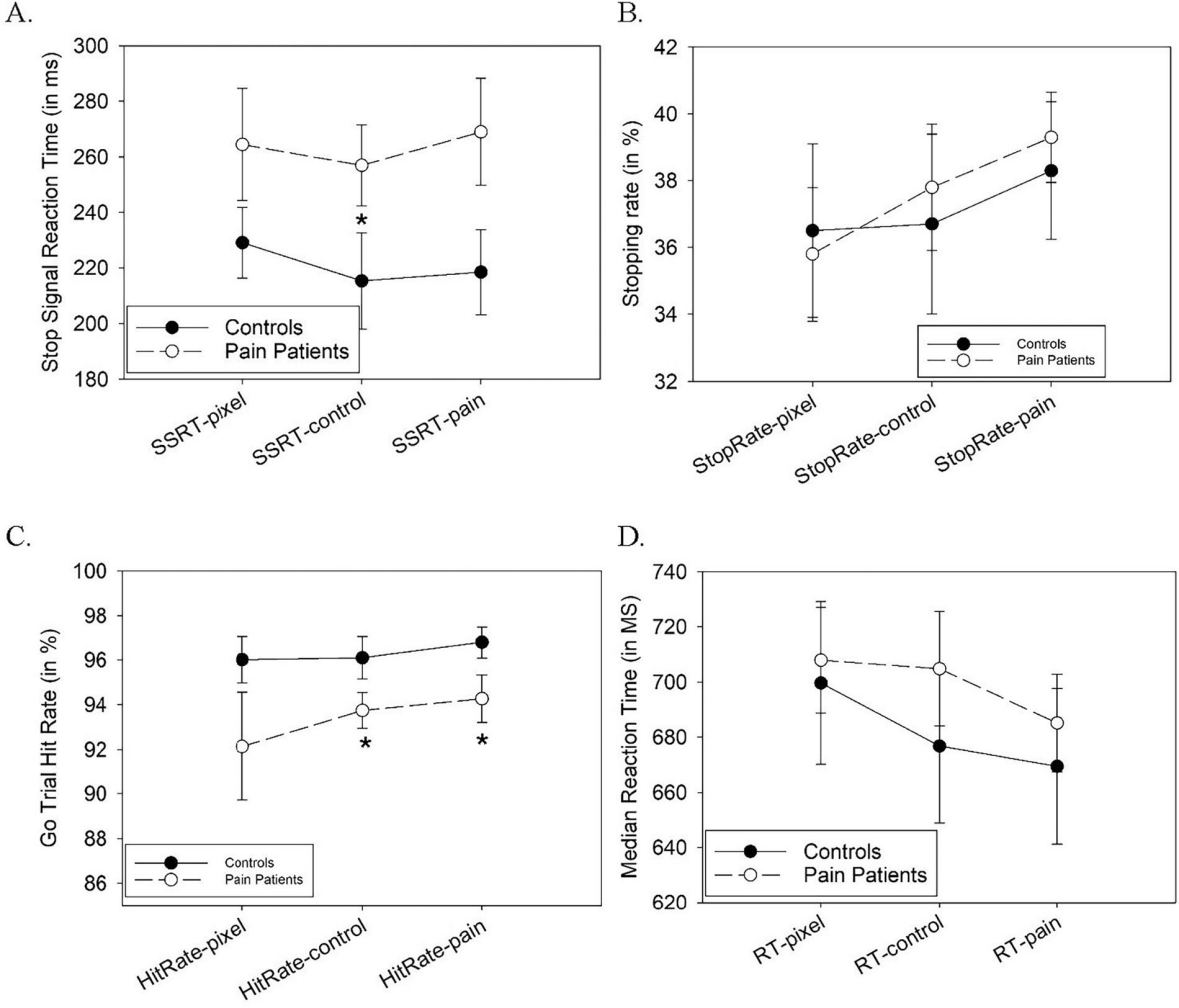
Behavior in the Stop Signal Task. Shown are: **(A)** stop signal reaction time (SSRT) values, **(B)** mean rates of successful stops in stop-signal trials under the three different background distractor conditions in each of the two groups (mean + SEM). Panels (**C)** and **(D)** show rates of accurate responses (hits) and reaction times, respectively, in the (predominant) “go” trials wherein a stop signal was not presented and the correct arrow directionality was reported. *Denotes significant group-wise difference *p* < .05.

In the SST, *n* = 17 CNMP Group participants and *n* = 14 Controls withheld responses in either <25% or >75% of stop-signal trials (suggestive of violation of the race model) and so were excluded from analysis (59). For example, very low response rates (i.e., high success rates) in stop-signal trials are indicative of a participant not complying with instructions to try to respond quickly and instead “waiting out” every trial to see if a stop signal appeared *before* initiating a motor response. Repeated-measures ANOVA indicated no main effect of background distracter type on stop-signal reaction time (SSRT) (*F*(2,65) = 0.203, *p* = .816) as the key behavioral impulsivity metric (Figure 2). There were also no effects of the background distractor images on rates of successfully withheld responses in stop trials (*F*(2,65) = 0.510, *p* = .602), or on either hit rates to targets in “go” trials (*F*(2,65) = 0.542, *p* = .584), or RT in “go” trials (*F*(2,65) = 0.450, *p* = .634). There were also no main or interaction effects of participant group in any of these metrics (all *p* > .600), with the exception of a trend toward a main effect of subject group on SSRT, where participants with CNMP showed a trend toward longer SSRT (across all three distracter conditions) than Controls (*F*(1,66) = 3.622, *p* = .061).

##### 3.2.1.2 Emotional go-nogo task (EGNGT)

**Figure 3:**
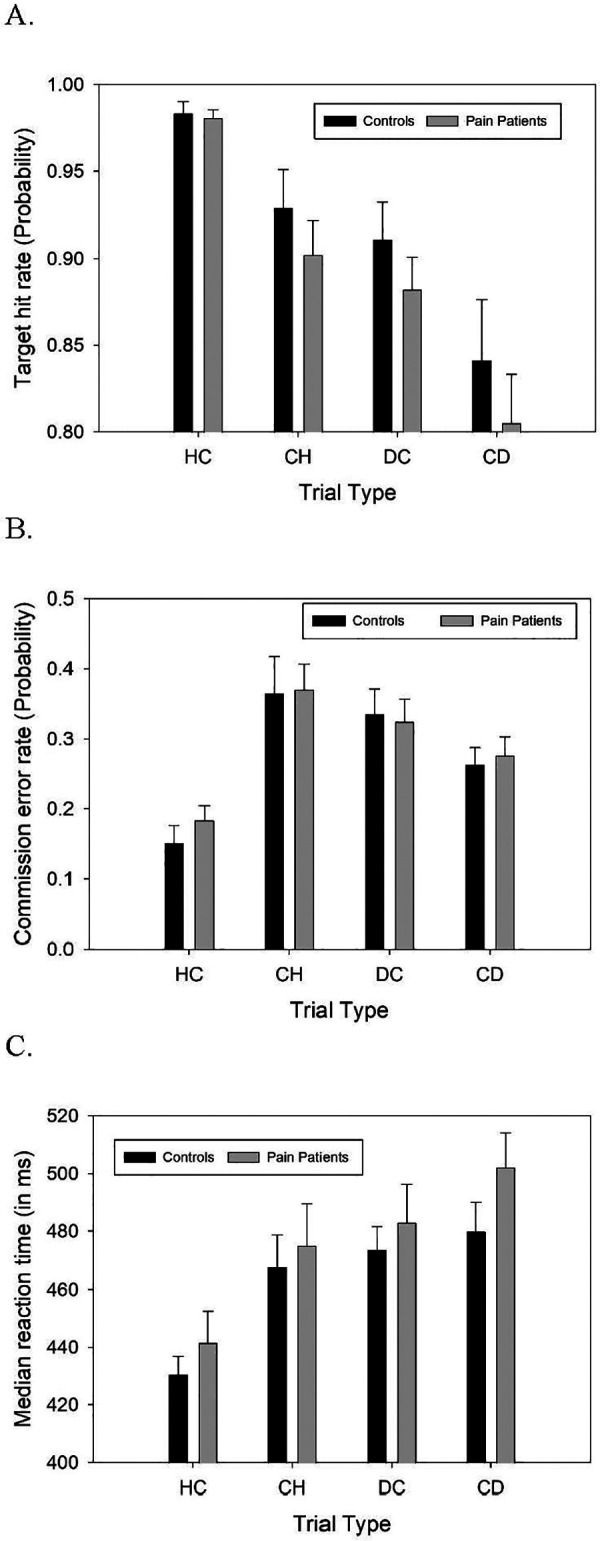
Behavior in the Emotional Go-NoGo Task (EGNGT). Shown are **(A)** hit rates*, **(B)** commission error (CE) rates, and **(C)** median reaction time (RT) to target images* in the EGNGT in each of the respond-happy, withhold-calm (HC) block, the respond-calm, withhold-happy (CH) block, the respond-distressed, withhold-calm (DC) block, and the respond-calm, withhold-distressed (CD) task block. There were no direct group differences in any metric in any trial type singly. *Y-axis range restricted for clarity.

In the EGNGT, data were not collected from one participant in the CNMP Group, and data were excluded for two other participants whose responding behavior indicated the opposite of the responding instruction for at least one task block. For hit rates, there were significant main effects of block valence (*F*(1,93) = 55.218, *p* < .001), where participants were more likely to respond correctly to targets when the block presented happy faces as either the targets (HC) or non-targets (CH), vs. blocks where distressed faces were the non-calm targets (DC) or non-targets (CD) (Figure 3A). There was also a main effect of the salience of target facial expression (*F*(1,93) = 18.417, *p* < .001), where hit rates were higher to behaviorally salient (happy/distressed) faces as targets relative to calm faces. There was no significant valence X salience interaction effect on hits, nor were there any significant main or interactive effects of participant group on hit rates (all *p* > .33).

For EGNGT commission error (CE) rates, there was a trend toward an effect of block valence (*F*(1,95) = 3.059, *p* = .084). Participants were more likely to respond inappropriately to nontargets when the block presented distressed faces as the non-calm faces (irrespective of whether the distressed faces were targets or nontargets, i.e., both DC and CD blocks), vs. blocks where happy faces were the non-calm faces (HC, CH blocks) (Figure 3B). There was also a significant effect of target face emotional salience (*F*(1,93) = 18.417, *p* < .001), wherein CE were more frequently elicited by distressed or happy faces than when nontarget faces were calm. A significant valence X salience effect (*F*(1,93) = 34.821, *p* = <.001) indicated that increased CE to emotional faces relative to calm faces was specific to distressed faces, whereas happy faces as non-targets reduced CE relative to calm faces as non-targets. However, there were no main or interactive effects of participant group on CE rates (all *p* > .550).

Analysis of RT in the EGNGT was limited to hit trials, due to the low incidence of CE. Median values for each trial type were used to minimize outlier effects of very long RT trials. There was a significant main effect of block valence (*F*(1,93) = 41.121, *p* < .001), where participants responded to targets faster when the block presented happy faces as the non-calm faces (i.e., HC, CH blocks), vs. blocks where distressed faces were the non-calm faces (DC,CD blocks) (Figure 3C). There was also a significant main effect of target emotional salience (*F*(1,93) = 17.948, *p* < .001, where RT was faster to happy/distressed faces than to calm faces. A significant valence X salience effect (*F*(1,93) = 5.123, *p* = 0.025) indicated that the effect of emotional faces as targets to speed RT was more pronounced for happy vs. distressed target faces. There were no main or interactive effects of group on RT. Similarly, there were no group differences in distress bias.

#### 3.2.2 Decision making tasks

##### 3.2.2.1 Delay discounting task (DDT)

**Figure 4:**
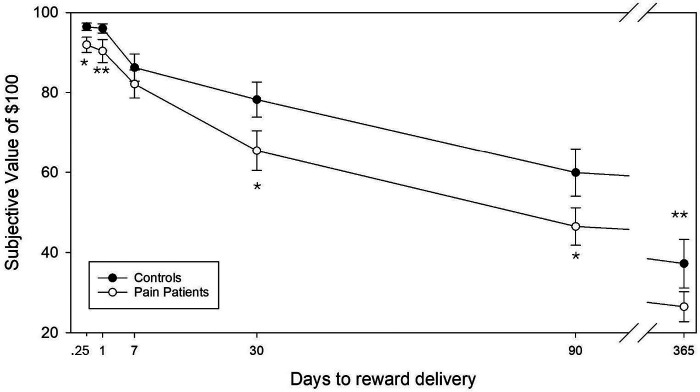
Behavior in the Delay Discounting Task. Shown are the hyperbolic patterns of discounting hypothetical $100 rewards at progressively longer delays to their receipt. Lines connect group-mean subjective values (SV) at different delay-based task blocks. The total area-under-curve of SV was significantly lower in the Pain Patient group, indicative of more severe discounting. For clarity, the 6 hr delay interval (.25 days) is not positioned at scale on the X axis, and the 5-year (1825 d) delay interval is not shown. **Denotes group difference at *p* < .10.

We excluded data from *n* = 24 participants of the CNMP Group and *n* = 6 Controls whose task choices did not show an orderly reduction of subjective value of rewards with increasing delay to delivery or who did not discount delayed rewards at all, per the two responding criteria of Johnson and Bickel for exclusion of disorderly data (72). These patterns may reflect the participant utilizing a simple responding heuristic (or responding randomly) rather than authentically deliberating on the value or desirability of the competing options in each trial. As shown in Figure 4, the CNMP Group devalued delayed rewards more severely than Controls with a significantly lower area-under-curve of subjective value (*t* = 2.147, *p* = .037), indicating greater decision-based impulsivity.

##### 3.2.2.2 BIRD frustration tolerance task

In the BIRD task, data were not available for one participant with CNMP and two Control participants. A slight majority of Pain Patients (31/60, 51.7%) and Controls (21/36, 58.3%) completed the final challenge task block without choosing to click on the stop button, with no difference in quitting between groups (Chi-sq 0.403, *p* = .527). Relatedly, there were no significant differences between the CNMP Group (mean 70.6 ± SD 96.8 s) and Controls (mean 111.8 s ± 124.8 s) in total time in challenge task before quitting. In the final challenge block, there was a trend toward a lower rate of mouse-click hits within the time window (i.e., birds “freed”) in the Pain Patients (mean 231.3 ± SD 82.7) compared to Controls (mean 264.6 ± 80.1) (*t* = 1.952, *p* = .055). However, after exclusion of three CNMP group participants' data due to outlying (> 3 SD from group mean) long RT values, RT to challenge targets was nearly identical in the CNMP Group (650.1 ± 93.0 ms) and Controls (648.2 ± 67.4 ms) (n.s.).

In repeated-measures ANOVA of self-reported mood ratings collected before and after the BIRD task, there were main effects of group (*F*(1,93) = 6.096, *p* = .015) and time (*F*(1,93) = 4.820, *p* = .031) on anxiety, where anxiety was elevated throughout in the CNMP Group and increased from pre- to post-testing in both groups (see Supplementary Figure S1) such that the group X time interaction effect was not significant. Self-reported frustration was also significantly higher in the CNMP Group (*F*(1,93) = 6.232, *p* = .014) and similarly increased from pre- to post-task (main effect of time *F*(1,93) = 26.014, *p* < .001) in both the CNMP Group and Controls, with no group X time interaction. The same was true for irritability, which was higher in CNMP Group (group effect *F*(1,93) = 4.283, *p* = .041) and increased from pre- to post-task in both groups (time effect *F*(1,93) = 22.006, *p* < .001), with no group X time interaction. Conversely, self-reported happiness was lower in the CNMP Group (group effect *F*(1,93) = 6.719, *p* = .011) and further decreased from pre- to post-testing (time effect *F*(1,93) = 5.106, *p* = .028), with a trend toward happiness reduction being more specific to CNMP (group X time effect *F*(1,93) = 2.649, *p* = .10).

#### 3.2.3 Implicit association task (IAT) modified with distress-related words

In the implicit association task (IAT), both groups responded accurately to the appropriate side designation/categorization for each presented word, with a trend toward lower accuracy in the CNMP Group (mean 92.5% ± SD 8.2) compared to Controls (mean 94.9% ± 5.8) (*t* = 1.685, *p* = .095). There were no group differences, however, in the self-positivity bias statistic “d”, where both the CNMP Group (mean *d* = .709 ± SD 0.38) and Controls (mean *d* = .725 ± SD 0.35) showed similar d-values indicative of a strong self-positivity bias (*t* = 0.223, *p* = .824).

### 3.3 Interrelationships between symptomatology scores

Across all participants, all interview-based questionnaire total and subscale scores intercorrelated with each other (all Pearson *r* ≥ . 45, all *p* < .0001) (see Supplementary Table S1). This supported our planned principal components analyses (PCA) to reduce correlations between symptomatology and task behavior. In our primary PCA, we included total scores on the PHQ-9, GAD-7, PCS, TSK, and PPI plus the Cognition subscale of the WHODAS (not all participants were eligible to complete the WHODAS Work/School activities scale). This PCA indicated that a single dominant factor (hereafter termed Distress Factor (DF)) accounted for 73.8% of the variance in symptomatology assessment scores, with the second factor accounting for only 10.6% of the variance (See Supplementary Figure S2 scree plot). In addition, due to the centrality of negative mood in pain disability and pain perception (26), we also performed a second, exploratory PCA that included solely the PHQ-9 and GAD-7, to relate behavior to mood disturbance specifically. This indicated that a single factor (hereafter termed Mood Factor (MF)) accounted for 90.5% of (shared) variance in PHQ-9 and GAD-7 scores.

### 3.4 Relationships between distress factor or mood factor and cognitive task performance

We wished to determine whether behavioral task metrics related directly to individual differences in distress, irrespective of group assignment. For those task assessments with a limited number of key outcome metrics, we conducted Spearman rank-order correlations across all participants. These analyses indicated no significant correlations between (six-assessment-based) DF scores in either: DDT area-under-curve of subjective values, IAT self-positivity bias, EGNGT commission error rates or EGNGT distress bias. There was also no statistically significant role of DF in predicting incidence of quitting (per logistic regression) or total time spent the final challenge block of the BIRD task. The two-assessment-based MF scores correlated positively with all other questionnaire scores singly (all Spearman *r* ≥ .640, *p* < .0001). MF scores also did not relate to EGNGT biases or commission errors, or BIRD task or IAT behavior, but showed a trend toward a negative correlation with area-under-curve in the DDT (Spearman *r* = −.23, *p* = .053), where higher MF factor scores (more mood disturbance) correlated with lower subjective value of delayed rewards (more severe discounting) (see Supplementary Figure S3).

Participants in the CNMP Group demonstrated a wide range of DF values, with only a subset showing DF values that would fall above the distribution of DF values of Controls (see Supplementary Figure S4). For more complex tasks, we conducted *post-hoc* analyses to determine whether anticipated behavioral differences between the CNMP Group and Controls might be uniquely detectable only in those CNMP participants who endorsed more severe distress. We subdivided the CNMP Group into two (sub)groups: participants whose DF score would fall within the primary distribution of DF scores of the Control group (Low-Distress Pain group, *n* = 16) vs. those with scores higher than the primary control distribution (High-Distress Pain group, *n* = 37). These subgroups did not differ in age (*t*-test *p* = .64) or sex representation (Chi-Sq *p* = .47). We then re-performed using three groups the mixed model ANOVAs of key response metrics in the SST and EGNGT across their different trial conditions.

Repeated-measures ANOVA of SST behavior with three groups indicated a main effect of (sub)group on SST (*F*(2,64) = 4.195, *p* = .019), wherein longer SSRT (more motoric impulsivity) was specific to the High-Distress Pain group (Supplementary Figure S5), with no differences between Controls and the Low-Distress Pain group. However, there were still no group X background distractor interaction effects on SSRT (all *p* > .5). We then performed complementary multiple regression analyses of behavior under each distractor condition separately, wherein primary group (CNMP vs. Control) and DF were concurrently entered as independent variables, and SSRT the dependent variable. Across all participants, DF scores showed a significant positive relationship with SSRT amid pain image backgrounds (i.e., after controlling for primary group assignment) (Beta = .449, *p* < .001; Supplementary Figure S6). Conversely, this independent relationship between SSRT and distress was not significant during control image (Beta = .216, *n.s.*) or pixelated (Beta = .185, *n.s.*) background conditions. In the EGNGT re-analysis with three groups, main and interaction effects of block valence and of face salience that were evident in the primary two-group analysis above remained for each of hit rates, commission error rates and median RT to targets, with no significant main or interaction effects of (subdivided) group on performance metrics.

## 4 Discussion

To obtain additional support for prevailing biopsychosocial models of chronic pain and to extend them to veterans, we administered to veteran outpatients being treated for CNMP a broad panoply of assessments, ranging from interview-based questionnaires on mood and other symptomatology, to neurobehavioral assessments of each of visual attentional bias, signal detection, decision-making, semantic bias, and frustration tolerance. Specifically, we wished to determine whether the elevated symptomatology and distress we expected to find in participants with CNMP was also directly linked to behavioral manifestations of attentional bias as a complementary subconscious manifestation of a mental preoccupation with pain. Specifically, we hypothesized that compared to Control veterans who neither endorsed CNMP nor received VHA care for CNMP, veterans with CNMP would show reduced frustration tolerance as well as exaggerated attentional capture by pain or discomfort-related stimuli, as evidenced in prolonged reaction time (RT) when viewing distress-related images in the EGNGT and by prolonged stop signal reaction time (SSRT) required to terminate an already-initiated motor response. This was predicated on an assumption that CNMP care-seeking would be prompted by or characteristic of greater psychological distress or perceived disability stemming from CNMP (50), and that these subjective experiences would be objectively behaviorally manifested in both exaggerated attentional capture by pain-related images or words (43, 73, 74). as well as in more severe delay discounting (33).

### 4.1 Psychological symptomatology

We replicated in a veteran sample previous findings of greater affective symptomatology and perceived disability in other chronic pain patients (75–77), where a mature literature has established that care utilization for chronic pain is linked to comorbid affective disorder (78, 79). Not only were scores on every assessment of symptomatology or pain-related distress significantly higher in the CNMP Group compared to Controls, scores of these instruments strongly intercorrelated with each other across all participants, such that most of the variance in scores on these assessments were captured in a single latent “distress” factor (DF). DF scores, as well as MF scores were also higher in the CNMP Group. That these different facets of psychological distress would intercorrelate so highly suggests a manifestation in a chronic pain context of a core metric or index of psychological burden generally. Studies of mental illness comorbidity, heritability and symptomatology over time within-subject have collectively shown that an omnibus factor that indexes overall psychological distress (“p” factor) accounts for substantial variance in all mental symptomatology (80). We surmise that high scorers on the primary (six-assessment DF) and exploratory (two-assessment MF) PCA factors possess an elevated *p* factor, where in a pain or injury context, this *p* factor would include pain catastrophizing and kinesiophobia.

### 4.2 Neurobehavioral findings

We found that each of the cognitive tasks administered showed generally orderly and expected behavior, such as trial-wise differences in behavior across trials within the task itself, as noted below.

#### 4.2.1 Delay discounting

We demonstrated for the first time in veterans more severe subjective devaluation of hypothetical rewards as a function of delay to their delivery in persons with CNMP compared to Controls (33), as a decision-based marker of increased impulsivity (81). In addition, a *post hoc* analysis detected a trend for more severe devaluation of delayed rewards to correlate with more severe mood symptomatology. Increased discounting may be another component of their greater general symptomatology in that more severe delay discounting has been found in several mental illnesses (82), and also shows genetic overlap with other mental illnesses, such a major depression (29). We thus posit that interrelationships between more severe delay discounting and more mood symptomatology would not only prompt immediate pain care seeking but also a strong desire for immediate relief (i.e., reward) wherein a pain patient insists on treatment options that are perceived to provide immediate relief (spinal injections or prescriptions for opioid analgesics) over treatment options that are safer, but would require a greater time investment (such as CBT or physical rehabilitation), and by extension, deferred pain relief.

Although the present study used a primarily basic science approach, the findings suggest that impulsivity may be an important characteristic among veterans experiencing pain, with potential clinical implications. Despite its relevance, management of impulsivity is actually not incorporated into most existing models of pain treatment (including CBT). However, it may play a mechanistic role in behaviors that reinforce pain and disability, particularly the avoidance of physical activity and the use of medication or other substances to manage discomfort (22). Avoiding physical or emotional distress can provide an immediate sense of safety or relief, even when engaging in rehabilitation—such as physical activity that may temporarily increase pain—would be more beneficial in the long term (i.e., discouting the ultimate good). This short-term reinforcement ultimately contributes to the persistence of pain and related disability (22). Ironically, although impulsivity is typically conceptualized in the cognitive domain, it is more directly targeted in treatments emphasizing emotion regulation, such as dialectical behavior therapy (83). Accordingly, there is both theoretical support and clinical precedent for integrating these types of regulatory skills into more comprehensive pain treatment approaches.

#### 4.2.2 Pain-related attentional bias

We found only mixed evidence that distress-related images captured attention specifically or more strongly in the CNMP Group. Unlike persons with opioid use disorder (62), participants with CNMP did not show exaggerated RT-based attentional bias when responding in EGNGT trials that featured distressed faces. Nor did pain patients emit more commission errors to non-target faces as a simple metric of motoric impulsivity. Participant behavior in the EGNGT instead merely replicated the previously reported tendency (in neurotypical individuals) for emotionally-salient faces as non-targets to elicit a greater frequency of commission error responses and for happy faces as targets to speed RT (61, 62). Re-analysis of distress bias or other trial type effects on RT or errors when Pain Patients were subdivided based on severity of psychological distress (DF scores) also did not reveal idiosyncratic response behavior in the High-Distress CNMP group, nor did bias metrics in the EGNGT correlate directly with DF scores.

In the modified implicit association task (IAT) that featured semantic (lexical) cues for distress, both participants with CNMP and Controls responded accurately and also responded more quickly to laterally categorize each target word when self-related and comfort-related words were to be endorsed on the same side of the screen (same response key) compared to when self-related and distress-related words were to be endorsed on the same side. The mean response bias metric “d” of both groups exceeded the value (.65) considered by the original IAT developers to indicate a strong bias (here toward self-positivity) in the task (84). However, the CNMP Group did not show a relatively degraded self-positivity bias relative to Controls ostensibly due to their greater self-identification with distress. Re-analysis with three groups also did not reveal an idiosyncratic bias in the High-Distress pain group, nor did “d” values correlate directly with DF scores. This stands in contrast to findings from a dot-probe task that featured distress-related words, wherein persons with CNMP took longer to respond when the dot appeared opposite the screen from a pain-related distractor (85) word. We surmise that the self-enhancement bias [wherein most individuals assume positive features about themselves (86)] commonly detected in implicit tasks with self-related pronouns is resistant to degradation by symptomatology experiences.

In our SST variant, we introduced operationally meaningless but potentially distracting images of persons in pain that could degrade cognitive task performance. However, our task provided more evidence for greater general motoric impulsivity in CNMP than evidence for pain-related attentional bias. In our main group comparison, the CNMP Group demonstrated a trend toward longer SSRT than controls [in replication of (87)]. This difference from controls was significant in the High-Distress subset of the CNMP Group. Longer SSRT is thought to be a marker of impaired motor inhibitory function, wherein the brain takes longer to terminate an already-initiated motor response (weaker “brakes”), and this has been found in ADHD (60).

We suggest that the increased SSRT in the High-Distress pain subgroup (and the related direct relationship between DF scores and SSRT across all participants) may be another manifestation of an elevated general psychopathology (p) factor. In seminal meta-analyses, reduced motoric self control (88) as well as blunted recruitment of neurocircuitry thought to govern motoric self control (89) have been reported in most mental illnesses. Importantly, motoric self-control and emotional regulation through cognitive re-appraisal have been shown to share a common neurocircuit substrate (90). We note that in *post hoc* multiple regression analyses, SSRT showed independent positive relationships with distress (DF) scores *during the pain distractor task condition* across all participants after controlling for primary patient group. This relationship was also positive but not statistically significant during control image and pixelated background conditions. It may be that the distractor images of persons in pain served to render the SST more sensitive to individual differences in motor inhibition ability (not bias).

#### 4.2.3 Frustration tolerance

The “BIRD” frustration tolerance task increased state metrics of negative affect in both groups from pre- to post-testing, with higher overall ratings of negative affect in Pain Patients. However, there were no interaction effects of time with group on mood. Behaviorally, there were no group differences (either using the original two groups or in the three-group follow-on re-analysis) in incidence of participants opting to respond on the button to terminate the final block that was rigged with impossibly fast visuomotor response requirements, where roughly half of participants in each group opted to terminate the task early. Across all participants, neither general distress (DF) nor mood symptomatology (MF) independently predicted quitting. This stands in contrast to previous findings wherein both decisions to quit (67) and time spent in the final challenge block (69) correlated with anxiety symptomatology. However, we note that in the experiment that introduced the BIRD task, increased internalizing symptomatology in quitters relative to non-quitters was specific to female participants (67) and our sample of Veterans was mostly male in both groups. Also, this cartoonized gamified version of the PASAT-C for youth may have somehow mitigated individual differences in how negative affect might drive frustration tolerance in adults.

### 4.3 Study limitations

Several limitations of our sample and design may have prevented better detection of attentional bias and other behavioral metrics. First, participants completed these tasks while comfortably seated in their home or in a quiet laboratory, with no preparatory psychological manipulations that may have increased pain salience, or stoked anxiety for pain. In contrast, in other studies, pre-assessment psychological manipulations, such as holding forth the prospect of a painful task or observing others perform a painful task, have been shown to affect subsequent pain experience [particularly among individuals with predispositions to elevated fear and catastrophizing (91–94)]. Second, participants in the CNMP group were selected for having no self-reported histories of opioid use nor records of VHA-issued prescription opioid analgesics. While this ostensibly minimized neurobehavioral confounds of opioid use and acute withdrawal, this exclusion may have selected for a less symptomatic or resilient population of pain patients, to in turn minimize differences from controls. Indeed, a mature literature has long linked chronic opioid analgesic use with greater perceived disability, mood problems, or distress [e.g., (9)]. However, our observed mean PCS (catastrophizing) score in the CNMP Group (25.3) is similar to if not slightly greater than that of mixed CNMP populations (95). Moreover, given the documented role of avoidance in opioid use—and the fact that opioids are often used as a maladaptive method of emotion regulation among individuals with pain (96)—the current findings highlighting the potential role of impulsivity may be particularly relevant to this population. We caution, however, that we did not toxicologically verify opioid abstinence. Third, although none of the Control participants had histories of VHA pain care nor self-reported “chronic pain” lasting three months or more, many had elevated symptomatology scores. Indeed, in accordance with findings that veterans who seek VHA health care tend to have more economic (97) and psychological (98) problems than veterans who do not, our Control group featured on average mild anxiety (mean GAD-7 score of 5.3), despite the exclusion of veterans with histories of any VHA mental health care from the Control group (which made recruiting this group very difficult). This likely minimized primary group differences. Fourth, previous experiments that detected exaggerated attentional capture by pain-related visual stimuli typically used cover tasks where the pain stimuli needed to be encoded more deeply to solve the task, such as when trying to discern subtle differences between scenes in a search task (7), or greater reliance on use of pain-related words (6, 99) that must be processed semantically. Conversely, the distractors in the SST were operationally irrelevant images. Also, we note that we had to eliminate several different cases from each of the behavioral task analyses due to disorderly data. Future similar experiments in this population may benefit from use of less complex tasks, especially if the pain population utilizes opioid medications. Finally, we note that we had to omit behavioral task data of several participants from analysis due to responding which failed to meet arbitrary (but literature-informed) performance thresholds. Future studies could deploy simpler task variants for this population with improved instruction techniques.

### 4.4 Conclusions and future directions

In conclusion, we replicated in veterans previous findings of greater psychological distress in CNMP as well as greater decision-based and motoric impulsivity in the more symptomatic CNMP patients, where a common latent factor accounted for most of the variance in distress or symptomatology across several domains, and where this distress factor was directly linked to impaired motoric behavioral control across all participants. We report here for the first time in opioid-free veterans findings that impulsivity (31), attentional biases (99), and other neurobehavioral features characteristic of individuals with CNMP tend to be specific to persons who report greater catastrophizing or psychological distress. Future experiments in veterans could explore individual differences in impulsivity and attentional bias as a function of chronic opioid use, and in the context of opioid discontinuation, with an eye toward impulsivity countermeasures more regularly found in emotion (dys)regulation psychological treatments and which are increasingly integrated into newer lines of pain interventions (100). Finally, this study further supports that psychological symptomatology is a likely driver of perceived disability from pain, such that well-resourced health systems like the VHA promote pain patient engagement with wrap-around mental health care.

## Data Availability

The raw data supporting the conclusions of this article will be made available by the authors, without undue reservation.
