# Supplementary material for: Impulsivity and pain attentional bias in veterans receiving care for chronic non-malignant pain
Source: Front Pain Res (Lausanne). 2026 Apr 23;7:1652567. doi: 10.3389/fpain.2026.1652567 (PMC13149377; doi:10.3389/fpain.2026.1652567)

Impulsivity and pain attentional bias in veterans receiving care for chronic non-malignant pain
Supplementary Material

Group differences in mood ratings before and after the BIRD frustration tolerance task

Shown in Figure S1 below are mean $\pm$ SEM visual analog scale ratings of each of anxiety, frustration, irritability and happiness (ratings made from 0-100 along a 100 mm line) collected from participants just prior to (pre) and immediately following the conclusion of the BIRD task (post). *Denotes significant group-wise difference at $P < .05$ with two-tailed independent t-test.

Figure S1.

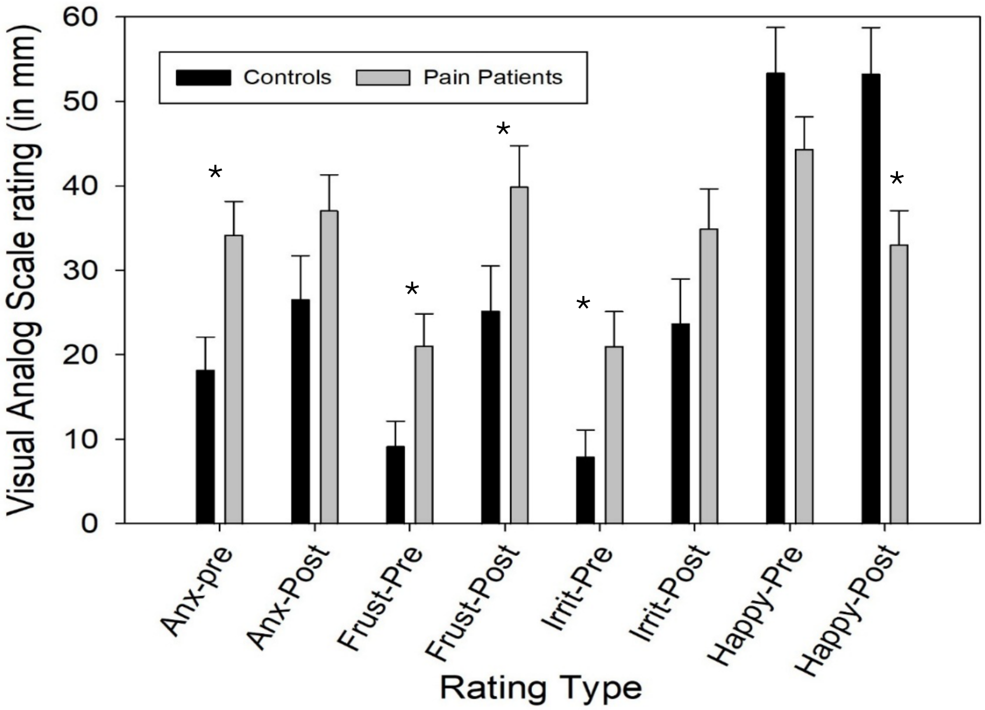

Interrelationships between symptomatology scores

Table S1 on the following page shows the bivariate Pearson correlation coefficients between total and subscale scores of self-report questionnaires (aside from PhenX toolkit Past-30-days substance use). All correlations are statistically significant to $p < .0001$. We therefore generated a planned principal components analysis (PCA) that incorporated total scores of the GAD-7, PHQ-9, PROMIS Pain Interference, Pain Catastrophizing Scale, and Tampa Scale for Kinesiophobia, along with the WHODAS Cognition subscale. A follow-on exploratory PCA composed of only PHQ-9 and GAD-7 scores yielded a common mood factor (MF) that captured 90.5% of shared variance in mood symptoms.

Table S1

Pearson Correlations

	GAD7	PCS_total score	PCS_Rumination	PCS_Magnification	PCS_Helplessness	PHQ9	PROMIS Pain Interf	TSK_total score	TSK_harm_somatic	TSK_avoidance	WHODAS -cognition	WHODAS Mobility	WHODAS Self-Care	WHODAS Getting Along	WHODAS Household Activities	WHODAS Work Activities
PCS_totalscore	.624**															
PCS_Rumination	.553**	.955**														
PCS_Magnification	.630**	.934**	.848**													
PCS_Helplessness	.616**	.976**	.891**	.879**												
PHQ9_SeverityScore	.810**	.684**	.642**	.644**	.674**											
PROMIS_Pain_Interference	.710**	.805**	.752**	.770**	.789**	.745**										
TSK_totalscore	.556**	.771**	.731**	.727**	.754**	.651**	.744**									
TSK_harm_somatic	.592**	.799**	.748**	.783**	.772**	.648**	.768**	.921**								
TSK_avoidance	.417**	.686**	.658**	.647**	.665**	.478**	.648**	.923**	.815**							
WHODAS_Cognition	.693**	.596**	.528**	.567**	.606**	.694**	.687**	.496**	.558**	.365**						
WHODAS_Mobility	.544**	.703**	.669**	.616**	.710**	.537**	.818**	.665**	.628**	.597**	.562**					
WHODAS_Self-Care	.637**	.616**	.559**	.535**	.643**	.671**	.707**	.575**	.524**	.440**	.633**	.742**				
WHODAS_Getting Along	.653**	.540**	.464**	.549**	.542**	.641**	.615**	.512**	.551**	.372**	.709**	.444**	.573**			
WHODAS_Household Activities	.646**	.690**	.626**	.653**	.692**	.701**	.784**	.647**	.628**	.542**	.637**	.766**	.719**	.533**		
WHODAS_Work Activities	.534**	.577**	.565**	.481**	.573**	.559**	.722**	.559**	.567**	.464**	.586**	.644**	.588**	.516**	.779**	
WHODAS_Participation	.790**	.697**	.615**	.705**	.690**	.767**	.807**	.570**	.623**	.440**	.762**	.689**	.748**	.748**	.802**	.804**

** Correlation is significant at $p < 0.0001$ level (2-tailed). Cells illuminated in yellow where $r > .5$

Figure S2 below shows the Scree plot from this PCA, wherein a single factor accounted for 73.8% of the variance in questionnaire scores.

Figure S2

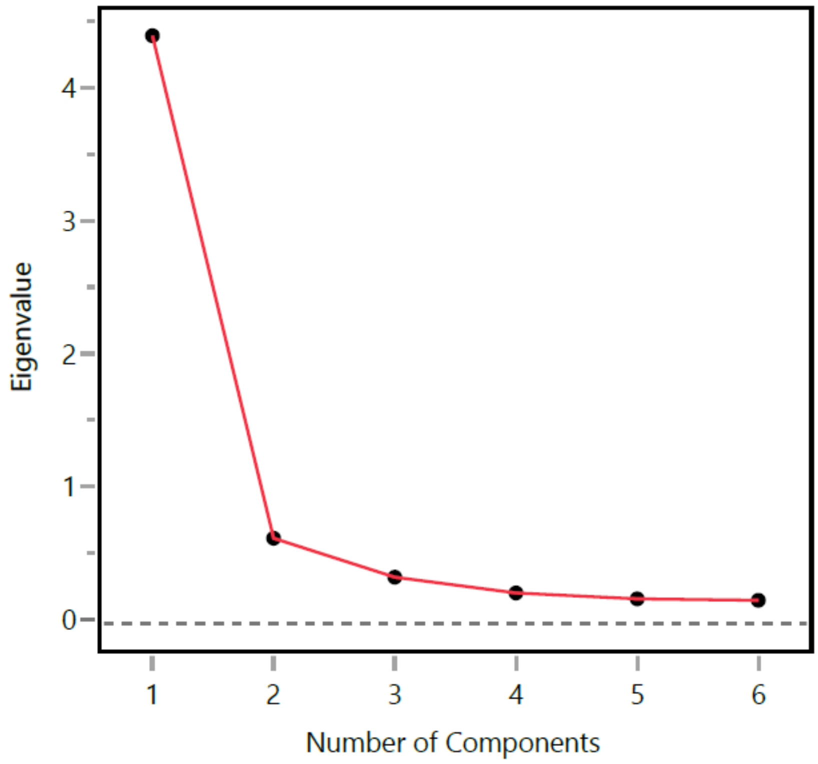

Re-analysis of direct relationships between psychological symptomatology and task behavior

The Mood Factor (MF) derived only from depression (PHQ-9) scores and anxiety (GAD-7) scores indicated a trend toward a negative correlation with area-under-curve in the DDT (Spearman r = -.23, p = .053), where higher mood symptoms correlated with lower subjective value of delayed rewards (Fig. S3):

Figure S3:

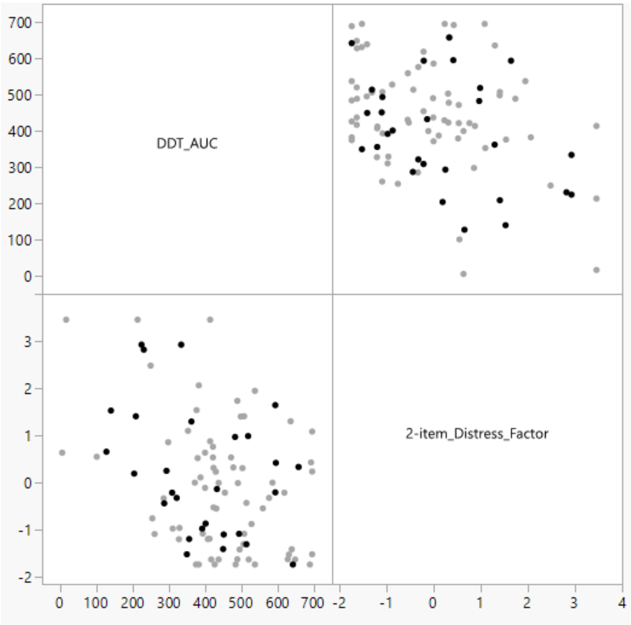

Formation of Pain Patient Subgroups

To isolate a discrete subset of pain patients who self-reported pronounced psychological distress such as from pain catastrophizing or depression, Pain Patients were subdivided into two groups based on having Distress Factor (DF) scores above the primary distribution of DF scores of the Control group (the Control distribution up through the Control participant with a DF value of .51 darkened black in Figure S4 below ((excluding the upper outlier)). Figure S4 below shows the distributions of individual DF scores (z-scaled with mean of 0 across all participants) between the two primary participant groups, and the further demarcation of the two Pain Patient subgroups.

Figure S4.

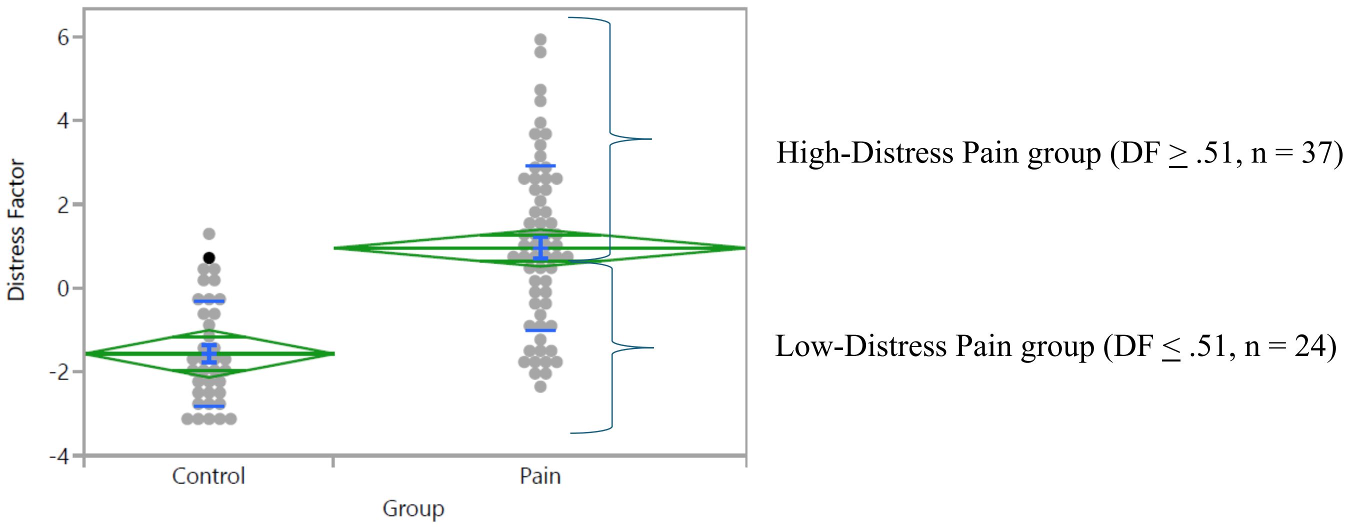

After this division of the Pain Patient group, the MANOVA of stop signal task behavior was re-performed as a three-group analysis after subdivision of Pain Patients into participants whose omnibus distress factor scores did vs not lie within the primary distribution of controls' distress factor scores (See Figure S2 above). Participants whose overall stopping rate was either less than 25% or greater than 75% were excluded, as in the primary two-group analysis. Figure S5 on the next page illustrates the pattern underlying a significant main effect of revised group on stop signal reaction time (SSRT) (across all three distractor conditions), wherein the High-Distress Pain group (n = 28) showed longer SSRT than the Low-Distress Pain group (n = 16) or Controls (n = 24). Wilcoxon rank-order tests indicated significantly slower SSRT in the High-Distress Pain group compared to both the Low-Distress Pain group and the Control group in the Pain-distractor trial condition, but this did not reach significance in the Control-distractor or Pixelated-distractor conditions.

Figure S5

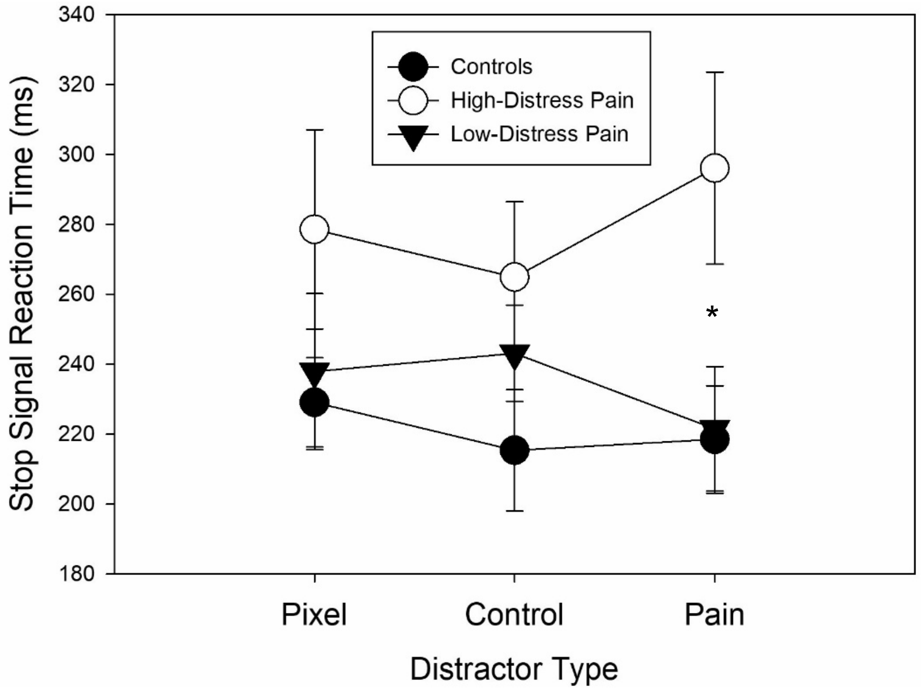

Second, we performed a complementary *post hoc* re-analysis of the relationship between symptomatology (distress factor (DF)) and SSRT using MANOVA, wherein original group assignment (Pain Patient vs Control) and DF scores were entered as independent variables, and SSRT the dependent variable. Analysis was conducted separately under each of the distractor conditions (pain, control, pixelated). This indicated that DF scores showed a significant positive relationship with SSRT under pain-image distractor conditions, wherein greater SSRT values (indicative of greater impulsivity) were found in participants with greater levels of overall distress (Beta = .449, *p* = .0012). See leverage plot below as Figure S6. However, this independent relationship between SSRT and distress was not significant during control image (Beta = .216, n.s.) or pixellated (Beta = .185, n.s.) background conditions.

Figure S6.

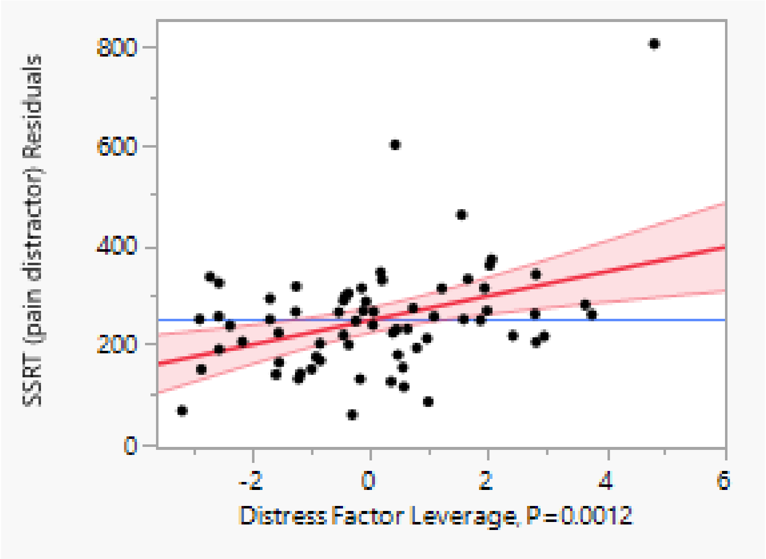

BIRD frustration tolerance task, time to quit in final task phase 3 among participants who quit

Shown in Figure S7 below are the distributions of time to quit (in milliseconds) for those participants who chose to quit the task early. After removal of the two upper extreme outliers of the Pain Patient group (orange box), the faster time to quit among Pain Patients was significant (two-tailed t = 1.477, p = .047).

Figure S7

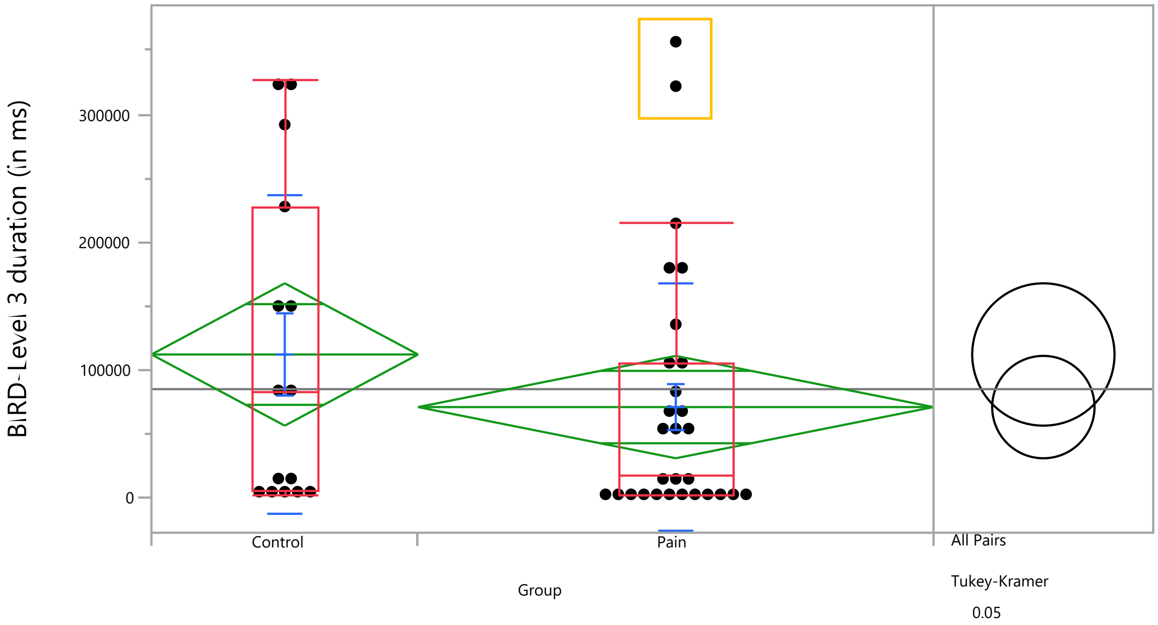

Supplement: Supplementary file 1 [file Datasheet1.pdf]
